# Immune Responses and Hypercoagulation in ERT for Pompe Disease Are Mutation and rhGAA Dose Dependent

**DOI:** 10.1371/journal.pone.0098336

**Published:** 2014-06-04

**Authors:** Sushrusha Nayak, Phillip A. Doerfler, Stacy L. Porvasnik, Denise D. Cloutier, Richie Khanna, Ken J. Valenzano, Roland W. Herzog, Barry J. Byrne

**Affiliations:** 1 Department of Pediatrics, Powell Gene Therapy Center, University of Florida, Gainesville, Florida, United States of America; 2 Department of Medicine, Center for Infection Medicine, Karolinska Institute, Stockholm, Sweden; 3 Amicus Therapeutics Inc., Cranbury, New Jersey, United States of America; 4 Department of Pediatrics, Cellular and Molecular Therapy, University of Florida, Gainesville, Florida, United States of America; University of Sao Paulo, Brazil

## Abstract

Enzyme replacement therapy (ERT) with recombinant human acid-α-glucosidase (rhGAA) is the only FDA approved therapy for Pompe disease. Without ERT, severely affected individuals (early onset) succumb to the disease within 2 years of life. A spectrum of disease severity and progression exists depending upon the type of mutation in the GAA gene (*GAA*), which in turn determines the amount of defective protein produced and its enzymatic activity. A large percent of the early onset patients are also cross reactive immunological material negative (CRIM-) and develop high titer immune responses to ERT with rhGAA. New insights from our studies in pre-clinical murine models reveal that the type of *Gaa* mutation has a profound effect on the immune responses mounted against ERT and the associated toxicities, including activation of clotting factors and disseminated intravascular coagulation (DIC). Additionally, the mouse strain affects outcomes, suggesting the influence of additional genetic components or modifiers. High doses of rhGAA (20 mg/kg) are currently required to achieve therapeutic benefit. Our studies indicate that lower enzyme doses reduce the antibody responses to rhGAA, reduce the incidence of immune toxicity and avoid ERT-associated anaphylaxis. Therefore, development of rhGAA with increased efficacy is warranted to limit immunotoxicities.

## Introduction

Pompe disease is an autosomal recessive, neuromuscular disorder caused by mutations in the gene encoding the lysosomal enzyme acid-α-glucosidase (GAA). GAA is required for the degradation of glycogen. Enzyme replacement therapy (ERT) with recombinant human GAA (rhGAA) is currently the only commercially available ameliorative therapy, however it is complicated by immune responses in severe early onset patients. Severely affected Pompe patients manifest symptoms as early as 1 month after birth with severe cardiomegaly, trouble feeding, poor muscle tone and respiratory distress [1], [2]. Without enzyme replacement, these patients do not survive beyond 2 years of age [3]. Pompe disease is characterized by a spectrum of manifestations. In addition to severe early onset, patients may exhibit juvenile onset or adult onset forms due to milder mutations [4]. The differences in manifestation and progression of the disease are dependent on the varying enzyme activity and levels of residual GAA. Presence of <1% GAA activity defines severe disease while >3% results in mild disease [4]. In the early onset forms, patients with no detectable cellular protein are designated as cross-reactive immunological material negative (CRIM-) [5]. CRIM+ patients have detectable, although insufficient or inefficient, protein and are less prone to immune reactions against rhGAA although some cases have been reported recently [6], [7]. Complete absence of GAA self-protein in the severe early onset patients causes their immune system to recognize the GAA epitopes as a non-self antigen. This identification leads to the onset of immune responses against the therapeutic rhGAA during ERT. The high doses of rhGAA (20 mg/kg to 40 mg/kg once every two weeks) required clinically can inadvertently trigger the immune system even in patients with mild mutations [7]. These high doses of rhGAA are required due to the inefficient uptake of rhGAA by cells [8]. Some patients develop infusion-associated reactions (IAR) such as sweating, headaches, elevated temperature or hypotension during infusions of rhGAA. Steroids and other anti-histamines like benadryl are often administered to prevent the occurrence of adverse events during rhGAA infusions in the clinic. In some cases, ERT has been discontinued due to the occurrence of these adverse reactions. Although allergy medications ameliorate symptoms of IAR, it is unlikely that they prevent binding of the antibody to GAA and the resultant treatment inefficacy. The possibility of adverse events occurring as a result of immune responses to rhGAA is an important factor during clinical trial design for Pompe disease. The rare incidence of Pompe (1∶40,000 live births) with mostly the early onset patients developing immune responses has resulted in insufficient study of immune responses to ERT [9]. Since few patients are available for study, the animal models of the disease for investigating these immune responses have become important. The *Gaa* knockout mouse has been observed to develop anti-GAA antibodies and anaphylactic responses to rhGAA in ERT [10]. We have bred the GAA-/- mouse on a pure 129SVE background (GAA-/- 129SVE) to provide a stable MHC background to facilitate immunological studies.[11]

Here we have begun to define parameters leading to immune responses and resulting physiological changes and toxicities occurring in response to rhGAA ERT. Specifically we have investigated the effect of the underlying GAA mutation (gene deletion vs. P545L missense mutation), the genetic background of the mouse (wild type (wt) BALB/c vs. wt 129SVE) and the dose of rhGAA. These studies lay the foundation for the future development of immune tolerance protocols for ERT and gene therapies. Furthermore, these data indicate the need for the development of a rhGAA enzyme with greater efficacy at reduced doses for the treatment of Pompe disease.

## Materials and Methods

### Mice

Wt BALB/c (known to be immunogenic in other protein deficiencies like hemophilia) and 129SVE mice (wt equivalent of the GAA-/-129SVE strain) were obtained from Jackson Laboratories (Bar Harbor, ME) and Taconic (Germantown, NY), respectively and were bred at the University of Florida. GAA-/- C57B/6 x 129SVJ mice were bred onto 129SVE background at Taconic (Germantown, NY) for greater than 10 generations to develop a pure strain ideal for immunological experiments.[11] The GAA-/- C57B/6 x 129SVJ strain was generated by insertional mutagenesis of exon 6 of the *Gaa* gene using a neomycin resistance gene [12]. P545L C57BL/6 transgenic mice expressing human GAA with a proline to leucine substitution at position 545 (Amicus Therapeutics. Cranbury, NJ) were bred onto GAA-/- 129SVE background mice and showed low GAA activity (data not shown). Both male and female mice were used in this study. The P545L mutation has been reported previously in humans [13], [14]. Humane endpoints were used in this study, loss of 15% of body weight from baseline weight or abnormalities persisting for over 24 hours like inactivity, labored breathing, sunken eyes or hunched posture or prolonged hypothermia were grounds for euthanasia. Animal experiments were approved by the University of Florida Institutional Animal Care and Use Committee (IACUC) under the protocol 201004611.

### Administration of rhGAA and Sample Collection

**Figure 1:**
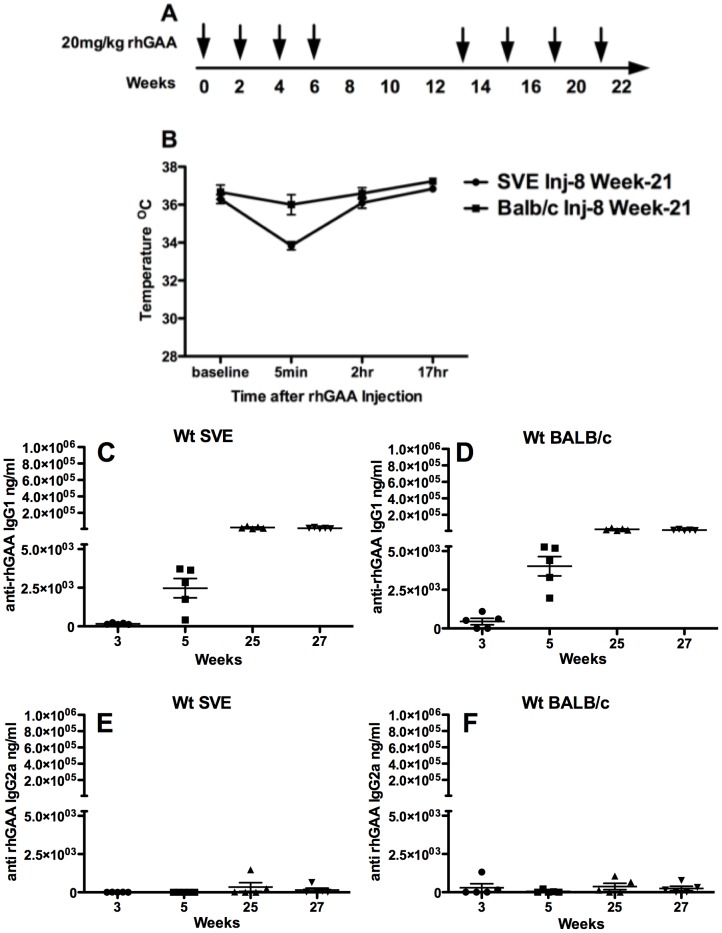
**A**) Experimental timeline **B**) Core temperature measurements prior to and post the 8^th^ rhGAA IV injection in wt 129 SVE and wt BALB/c mice (n = 5) **C**) Anti-rhGAA IgG1 antibody in 129SVE wt mice **D**) Anti-rhGAA IgG1 antibody in BALB/c mice **E**) Anti-rhGAA IgG2a antibody in wt 129SVE mice **F**) Anti-rhGAA IgG2a antibody in wt BALB/c mice.

Wt BALB/c, wt 129SVE and P545L C57BL/6x129SVE mice were injected intravenously (IV) via tail-vein with 20 mg/kg rhGAA (Myozyme, Genzyme, MA) once every two weeks for eight weeks and re-challenged again ∼eight weeks later, once every two weeks for an additional eight weeks (**Figure 1 A**) in an attempt to elicit a more severe immune response. GAA-/- 129SVE mice were injected once every two weeks with 1 mg/kg, 5 mg/kg or 20 mg/kg for up to 10 weeks. All rhGAA doses were calculated on mouse weight of 25 g. Blood samples were obtained by tail-vein or retro-orbital into an anti-coagulant coated tube (heparin or 3.8% sodium citrate) for enzyme linked immunosorbent assay (ELISA), complete blood count (CBC), iSTAT and clotting assays.

### Pulse Oxymetry

Measurement of vital signs like oxygen saturation, heart rate, pulse distention, breath distention and breath rate was done using a cardiopulmonary data recorder MouseOx pulse oxymeter (Starr Lifescience Corp. PA) as published previously [15], [16], [17]. Mice were anesthetized with isoflurane for all measurements. Baseline measurements were done first, after which mice were allowed to recover; rhGAA was then administered via tail-vein injection and further pulse oxymetry measurements were taken ∼5 minutes after the rhGAA IV injection, under anesthesia. Core body temperature was measured using a thermocouple thermometer.

### Bleeding Time

The time required for platelet plug/clot formation to occur after ∼1 mm tail-snip was determined. The injured tail was immersed in 37°C pre-warmed PBS, while the mice were anesthetized with 2% isoflurane and time taken for bleeding to completely stop was observed. Cauterization of the injured tail was performed at a maximum of 3 minutes after the tail-snip. Baseline bleeding time was measured prior to rhGAA injection and final bleeding times were measured 3 minutes after rhGAA IV injection.

### Activated Partial Thromboplastin Time (aPTT)

Standard aPTT tests were done at a 1∶2 dilution in aPTT reagent; aPTT was measured in the plasma using a fibrometer (Fibrosystem; BBL, Cockeysville, MD USA).

### ELISA

Anti-rhGAA IgG1, IgG2a, IgG2b and IgM assays were developed and optimized. Immulon 4HBX 96-well plates (Thermo-Scientific) were coated with rhGAA protein and incubated overnight at 4^ο^C. The following standards- IgG1κ (4000 ng/mL–62.5 ng/mL), IgG2a and IgG2b (1000 ng/mL–15 ng/mL), IgM (400 ng/mL – 6.25 ng/mL) were coated overnight at 4^ο^C at 2-fold dilutions. Experimental mouse plasma at a 1:50 dilution was used for IgG1, IgG2a and IgG2b ELISA while 1:20 dilution was used for IgM ELISA. Plasma samples were incubated for 2 hours at room temperature. The secondary detection antibodies rat anti-mouse IgG1 heavy chain-HRP (AbD Serotec, UK), goat anti-mouse IgG2a- HRP (Abcam, MA), goat anti-mouse IgG2b-HRP (Abcam, MA) or rat anti-mouse IgM-HRP (SouthernBiotech, AL) were incubated for 2 hours at 37^ο^C. Plates were allowed to develop for 5 to10 minutes in a solution containing Sigmafast OPD tablets (Sigma, MO) for color production. Plates were washed three times between procedures with tris wash buffer. Total IgE was measured using OptEIA mouse IgE ELISA Kit (BD Bioscience, CA). A colorimetric plate reader (BD Biosciences, San Jose, CA) was used to read the 96-well clear ELISA plates.

### D-Dimer Assay

D-Dimer in plasma (blood was collected via retro-orbital bleed in 3.8% sodium citrate) was quantified using Asserachrom D-Di enzyme immunoassay kit (Diagnostica Stago, Asnières, France) following the manufacturer's protocol. Samples were read at 450 nm using a µQuant microplate spectrophotometer (Bio-Tek Instruments, Winooski, VT).

### Histochemistry

Blood smears from naïve GAA-/- 129SVE mice or cohorts challenged with rhGAA were stained with eosin and methylene blue for platelet clump determination at the Animal Care Services lab (University of Florida, FL). Mouse tissues (n = 3) from naïve and IV rhGAA challenged mice (liver, heart and kidney) were preserved in 4% paraformaldehyde. Tissue sectioning and staining with eosin and hematoxylin was performed by the Molecular Pathology Core, University of Florida. Fibrinogen staining of paraffin sections was performed at the pathology core at University of Pennsylvania, utilizing rabbit anti-human fibrinogen primary antibody (Dako, Denmark) and anti-rabbit peroxidase Vectastain ABC kit (Vector Laboratories, Burlingame, CA). Images were analyzed using ImageScope software (v11.2.0.780; Aperio; Vista, CA) and are representative of 3 mice per group at a 5x magnification.

### Statistical Analysis

GraphPad Prism and Microsoft Excel software were used to determine unpaired two-tailed student t-test. P values <0.05 were considered to be statistically significant and results are represented as means ± standard error (SEM).

## Results

### Antibody responses to rhGAA ERT in wt 129SVE and wt BALB/c mouse strains

Wt BALB/c (n = 5) and 129SVE mice (n = 5) injected once in every 2 weeks with 20 mg/kg rhGAA for 8 weeks and re-challenged for an additional 8 weeks showed no outward signs of distress. Core body temperature in 129SVE mice measured after the tail-vein injection at week-21 showed an average 2^ο^C drop, 5 minutes after rhGAA injection; all animals recovered their normal body temperature within 2 hours (**Figure 1 B**). Anti-rhGAA IgG1 antibody increased from baseline to a high of ∼2×10^5^ ng/mL after the 8^th^ IV injection in both wt 129SVE and wt BALB/c strains (**Figure 1 C, D**). IgG2a responses were largely absent in both mouse strains (**Figure 1 E, F**). These antibody responses were low in comparison to the immune responses in the Pompe mice with the null or missense mutations (**Figure 3 D, H, L, P**). All wt 129SVE and wt BALB/c mice survived after the 8-week rhGAA challenge and 8-week re-challenge.

### Pulse oxymetric measurement of vital parameters pre- and post-rhGAA ERT in wt mice

**Figure 2:**
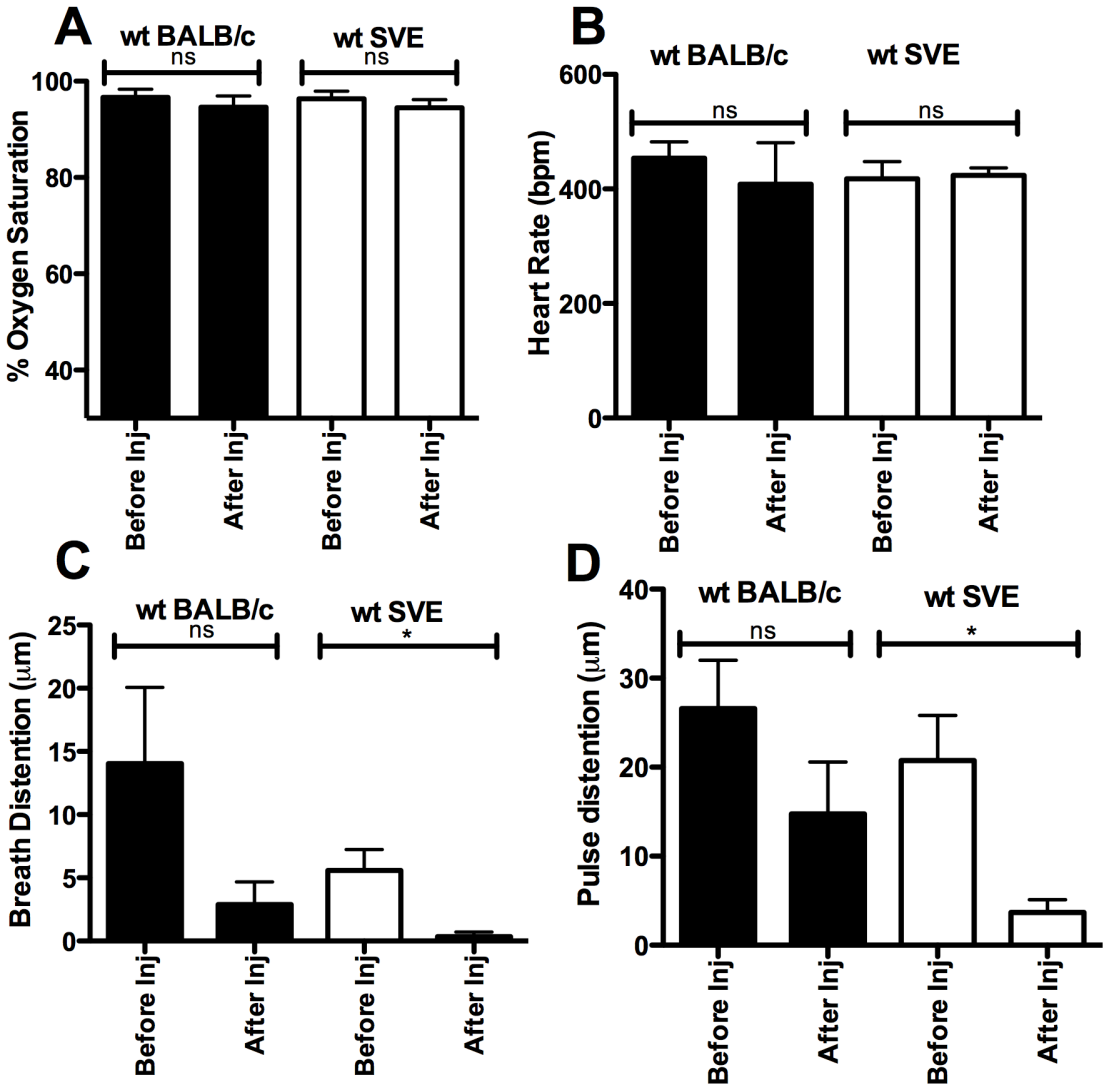
Vital signs measured by pulse oxymetry, prior to and 5 minutes after the 8^th^ rhGAA IV injection in wt BALB/c and wt 129SVJ mice (n = 5). **A**) Percentage oxygen saturation **B**) Heart rate **C**) Breath distention **D**) Pulse distention, p<0.05 *, ns = not significant, (inj; injection).

Vital parameters including oxygen saturation, heart rate, pulse distention (measures distention of the blood vessels under the pulse oxymetry probe and is indicative of blood flow) [17], breath distention (indirect non-invasive indication of the effort required by a subject to generate a breath) [15], [16] and breath rate were measured prior to and 5 minutes after the 8^th^ rhGAA (20mg/kg) injection in wt BALB/c (n = 5) and wt 129SVE mice (n = 5). These vital parameters are expected to be affected during the occurrence of anaphylaxis [18]. Change in oxygen saturation was not significant and remained at 94-96% pre- and post-rhGAA injection in both wt strains of mice (**Figure 2 A**), indicating normal levels of oxygen saturation. Heart rate and breath rate changes were within the normal ranges and there were no significant changes before ERT or 5 minutes post-ERT in either mouse strain (**Figure 2 B** **and data not shown**). Pulse distention showed a drop in some wt BALB/c mice with a pre-ERT average of 26 µm to a post-injection (8^th^ IV) average of 15 µm (**Figure 2 D**). The change in pulse distention was significant in the SVE mice with a drop from 21 µm to 4 µm (**Figure 2 D**). The reduction in breath distention values was not significant in the BALB/c strain while it was significant in the 129SVE strain (**Figure 2 C** **)**. Although significant, these changes were not accompanied by the severe immune responses and distress that were observed in the GAA-/- 129SVE and GAA-/- BALB/c x 129SVE Pompe mouse strains within 3 to 5 rhGAA tail-vein injections of 20 mg/kg rhGAA.

### Antibody responses to rhGAA ERT are antigen dose-dependent

**Figure 3:**
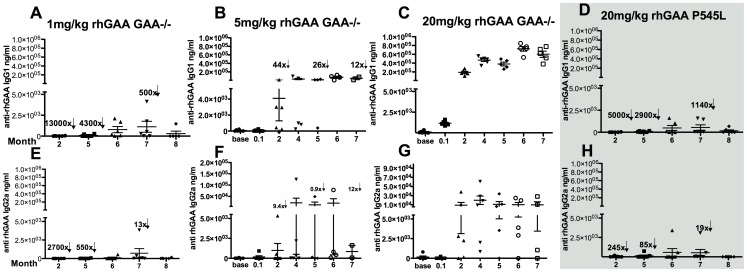
Antibody responses to varying doses of rhGAA in null mutation (n = 6) or P545L mutant mice (n = 5). **A**) Anti-rhGAA IgG1 in 1 mg/kg rhGAA injected GAA-/- 129SVE mice tested weekly **B**) Anti-rhGAA IgG1 in 5 mg/kg rhGAA injected GAA-/- 129SVE mice **C**) Anti-rhGAA IgG1 in 20 mg/kg rhGAA injected GAA-/- 129SVE mice **D**) Anti-rhGAA IgG1 response in 20 mg/kg rhGAA injected P545L mice **E**) Anti-rhGAA IgG2a in 1 mg/kg rhGAA injected GAA-/- 129SVE mice **F**) Anti-rhGAA IgG2a in 5 mg/kg rhGAA injected GAA-/- 129SVE mice **G**) Anti-rhGAA IgG2a in 20 mg/kg rhGAA injected GAA-/- 129SVE mice **H**) Anti-rhGAA IgG2a in 20 mg/kg rhGAA injected P545L mice. Arrows indicate fold decrease over corresponding 20 mg/kg cohort time point. p<0.05 *, p<0.005 **, p<0.0005 ***, ns = not significant.

GAA-/- 129SVE mice (n = 6) were administered 1 mg/kg, 5 mg/kg or 20 mg/kg rhGAA by tail-vein injection once every two weeks for up to 10 weeks. Re-challenge was not possible due to anaphylaxis and high mortality by the third or fourth rhGAA dose in the 5 and 20 mg/kg rhGAA cohorts. Plasma anti-rhGAA IgG1 antibodies (indicative of a Th2 response) increased from background levels (prior to ERT) up to an average of 1100 ng/mL in the 1 mg/kg rhGAA cohort (lowest observed response among the various dose cohorts, **Figure 3 A**). Anti-rhGAA IgG1 antibodies increased to an average of 74000 ng/mL and 724000 ng/mL (**Figure 3 B, C**) i.e. 67-fold higher in the 5 mg/kg cohort and 650-fold in the 20 m/kg cohort respectively, in comparison to the 1 mg/kg low dose cohort. Similarly, a rise in IgG2a response (Th1) was observed with the 1 mg/kg cohort, rising to an average of 715 ng/mL, the 5 mg/kg cohort increased to 21200 ng/mL (with one high response outlier) and the 20 mg/kg cohort showed highest consistent increase to 19950 ng/mL, with all mice producing a prolonged high IgG2a response (**Figure 3 E-G**). Increases in IgG2b were also observed, with the 1 mg/kg cohort indicating a response average of up to 100 ng/mL with one mouse outlier showing 6974 ng/mL. The 5 mg/kg rhGAA cohort had up to a 75 ng/mL average and the 20 mg/kg cohort had up to a 1076 ng/mL average IgG2b response (**Figure S1 A-C**). An increase in IgM levels was observed as early as 1 week after rhGAA administration, with the 1 mg/kg cohort increasing to up to 420 ng/mL, the 5 mg/kg cohort increasing to an average of 1400 ng/mL and the 20 mg/kg cohort rising to an average of 1800 ng/mL (**Figure S1 E-G**).

### Mutation type influences the anti-rhGAA immune response

P545L Pompe mice were administered 20 mg/kg rhGAA once every two weeks for up to 10 weeks. IgG1 responses rose from background up to 536 ng/mL and were 870-fold lower than the 20 mg/kg treated GAA-/- 129SVE mice, 7-fold lower than the 5 mg/kg treated GAA-/- 129SVE mice and 2-fold lower than the lowest dose cohort 1 mg/kg treated GAA-/- 129SVE mice (**Figure 3 D, A** **).** Similarly, IgG2a responses in the P545L mice increased up to 580 ng/mL (**Figure 3 H**) and were 34-fold lower than the 20 mg/kg GAA-/- 129SVE group, 36-fold lower than the 5 mg/kg GAA-/- 129SVE group (**Figure 3 F, G**) and comparable to the 1 mg/kg rhGAA treated GAA-/- 129SVE cohort (**Figure 3 E**). IgG2b increased to 123 ng/mL (**Figure S1 D**) and was 8-fold lower than the 20 mg/kg group, 2-fold lower than the 5 mg/kg group and comparable to the 1 mg/kg treated GAA-/- 129SVE group (**Figure S1 A-C**). IgM levels were also at least 3-fold lower than the 5 mg and 20 mg/kg GAA-/- 129SVE treated cohorts at 575 ng/mL although one outlier was observed at 4233 ng/mL (**Figure S1 H**).

### Core temperature and mortality are affected by rhGAA dose and mutation type

**Figure 4:**
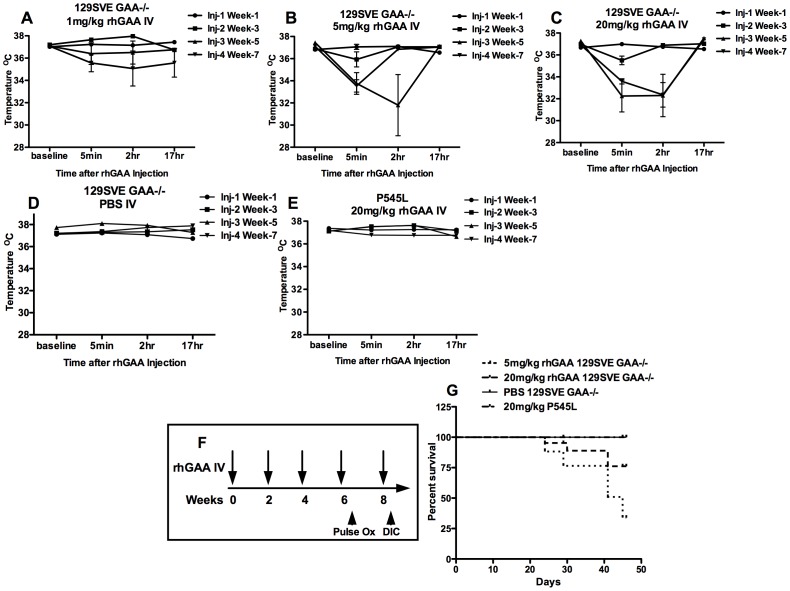
Temperature measurements prior to and post rhGAA IV injections in A-C) GAA-/-129SVE mice (n = 6) receiving 1 mg/kg, 5 mg/kg or 20 mg/kg doses of rhGAA IV D) GAA-/-129SVE mice injected with PBS E) P545L mice (n = 5) receiving 20 mg/kg of rhGAA IV, F) Experimental timeline indicating GAA-/- 129SVE mice or P545L C57BL/6 x 129SVE mice injected with 1 mg/kg, 5 mg/kg or 20 mg/kg doses of rhGAA G) Survival curve.

Core temperatures were measured prior to every rhGAA tail-vein injection followed by measurements at 5 minutes, 2 hours and 17 hours after each rhGAA injection. GAA-/- 129SVE mice (n = 6) were injected with 1 mg/kg, 5 mg/kg or 20 mg/kg rhGAA by tail-vein. A drop in core body temperature was observed in the 1 mg/kg, 5 mg/kg and 20 mg/kg GAA-/- 129SVE cohorts (**Figure 4 A, B, C**). The drop in core temperature was progressively worse with every additional rhGAA administration and mice began to show severe anaphylactic responses by the 3^rd^ injection. Mice injected with the 5 mg/kg rhGAA dose showed a more severe temperature drop than the 20 mg/kg dosed mice, two out of six mice from this group died by the 3^rd^ rhGAA injection (**Figure 4 F, G**). Control GAA-/- 129SVE mice injected IV with PBS had normal core temperatures after each injection (**Figure 4 D**). Mice carrying the P545L missense mutation continued to show normal core temperature after multiple 20 mg/kg rhGAA injections and no observed anaphylactic distress like piloerection or labored breathing (**Figure 4 E**). These results can be compared with wt BALB/c mice that did not show any temperature changes after rhGAA injections, while wt SVE mice showed a two-degree drop in temperature after the 7^th^ injection that was not accompanied by any visible signs of anaphylactic distress (**Figure 1 B**).

### Anaphylactic sensitization to rhGAA is dose-dependent and affected by mutation type

**Figure 5:**
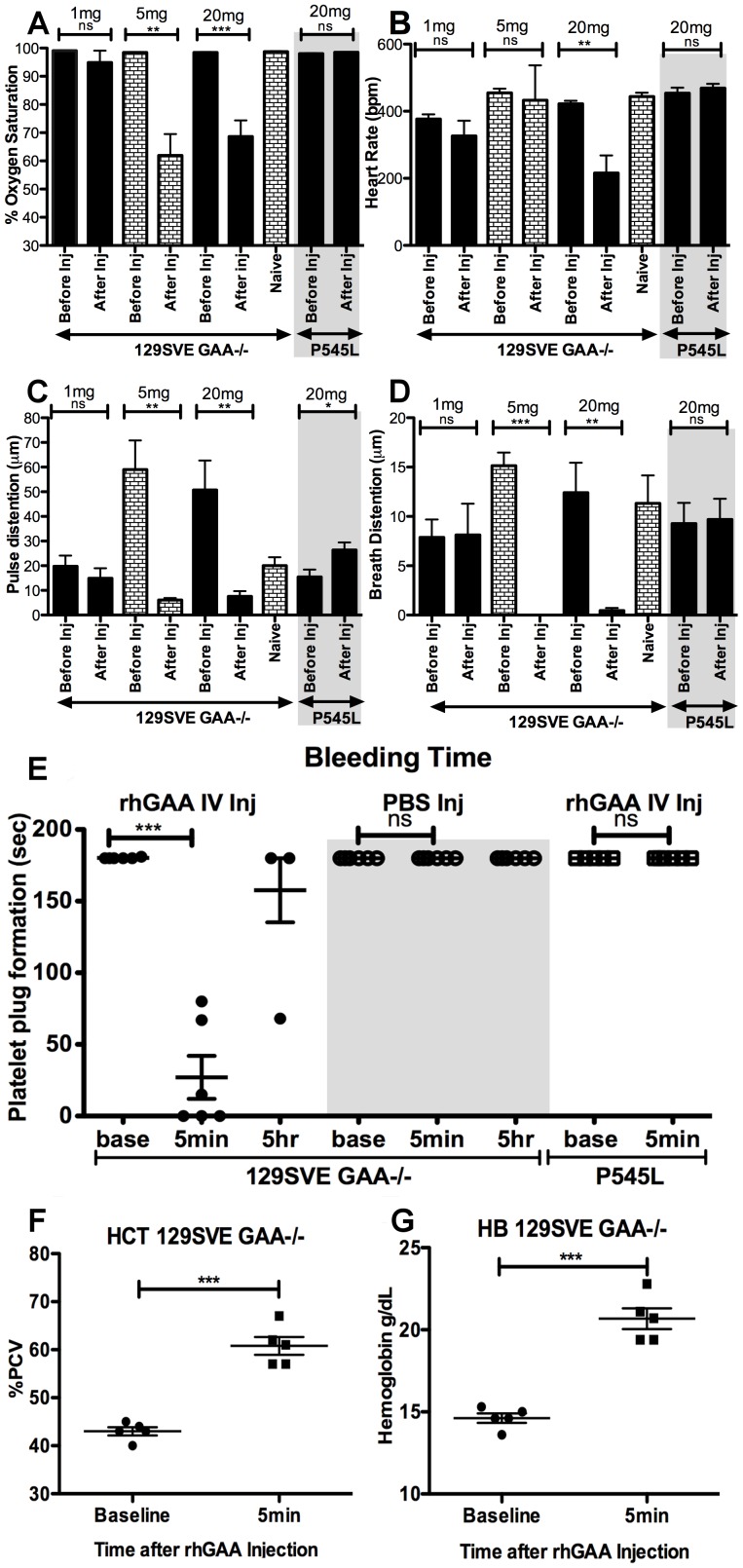
Comparison of pulse oxymetry measurements of vital signs prior to and post the 4^th^ rhGAA (1 mg/kg, 5 mg/kg or 20 mg/kg) ERT IV injection in GAA-/- 129SVE (n = 6) and P545L mice (20 mg/kg rhGAA; n = 5). A) oxygen saturation B) heart rate C) pulse distention D) breath distention, E) Time taken for the formation of a platelet plug post-injury (tail-snip) prior to and post-rhGAA IV administration. Changes in hemorheologic values from complete blood counts in 129SVE GAA-/- mice and P545L naive and 20 mg/kg rhGAA injected mice (5 min post rhGAA injection). F) Hematocrit, G) Hemoglobin, p<0.05 *, p<0.005 **, p<0.0005 ***, ns = not significant.

Pulse oxymetry was used to measure arterial blood oxygen saturation in the GAA-/- 129SVE and P545L mice as an indication of the percentage of hemoglobin saturated with oxygen. A significant rhGAA dose-dependent drop in oxygen saturation was observed in GAA-/- 129SVE mice (n = 6) injected with 5 mg/kg rhGAA (98% to <60%) or from 98% to ∼65% oxygen saturation for the 20 mg/kg group when measured immediately before the rhGAA IV injection and approximately 5 minutes after the third and fourth rhGAA tail-vein injections (**Figure 5 A**). An additional group treated IV with 1 mg/kg rhGAA did not show a notable change in oxygen saturation (**Figure 5 A**). Furthermore, mice carrying the GAA P545L mutation administered with 20 mg/kg rhGAA did not show a change in oxygen saturation prior to and ∼5 minutes after rhGAA tail-vein injection (**Figure 5 A**). PBS-injected GAA-/- 129SVE mice had no notable change in arterial oxygen saturation, indicating not only the dose dependence of the physiological responses to rhGAA ERT but also its dependence on the type of mutation.

Heart rate was measured in the 1 mg/kg and 5 mg/kg dose cohorts and no change prior to and ∼5 minutes after the rhGAA injection was observed (**Figure 5 B**). In the 20 mg/kg dose cohort, a significant drop in heart rate was observed from 400bpm prior to rhGAA injection to 200bpm after the injection (**Figure 5 B**). 20 mg/kg rhGAA treated P545L mice and GAA-/- 129SVE mice injected with PBS did not show a drop in heart rate (**Figure 5 B** and data not shown).

Pulse distention measures distention of the blood vessels, indicative of blood flow [17]. A severe reduction was observed 5 minutes after the fourth rhGAA IV injection of 5 mg/kg or 20 mg/kg in GAA-/- 129SVE mice (**Figure 5 C**) while 1 mg/kg dosed mice showed a small reduction in pulse distention (**Figure 5 **C). The pulse distention in the 5 mg/kg and 20 mg/kg rhGAA administered mice was unusually high even prior to the 5^th^ rhGAA injection (**Figure 5 C**). P545L mice injected with 20 mg/kg rhGAA and PBS injected GAA-/- 129SVE mice showed a slight increase in pulse distention 5 minutes after injection, which may be expected due to the use of a heat lamp during the procedure of rhGAA injection (**Figure 5 C**) and was similar to a group injected with PBS (data not shown).

Breath distention is a non-invasive indication of the effort required by a subject to generate a breath [15], [16]. Pulse oxymetry measurements showed a precipitous drop in breath distention that was more severe in 5 mg/kg injected than 20 mg/kg injected GAA-/- 129SVE mice ∼5 minutes after injection (**Figure 5 D**). There was no drop in breath distention in the 1 mg/kg rhGAA cohort (**Figure 5 D**). P545L mutant mice also did not show a drop in breath distention upon rhGAA injection (**Figure 5 D**). Breath rate was largely unaffected by the administration of rhGAA in the PBS, 1 mg/kg and 5 mg/kg GAA-/- 129SVE cohorts; however, there was a significant drop in breath rate in the 20 mg/kg rhGAA group (data not shown). In the P545L group injected with 20 mg/kg rhGAA, the breath rate remained stable (data not shown).

### Physiochemical changes to multiple antigen exposures in GAA-/- 129SVE mice

A significant increase in hematocrit levels (HCT) was observed 5 minutes after injection with rhGAA. HCT were measured in venous blood by complete blood count and using an iSTAT (Abaxis, Union City, CA). Lactate levels were abnormal prior to rhGAA administration due to the underlying Pompe disease condition and did not change immediately after rhGAA injection (data not shown). Increases in HCT are often representative of increased blood viscosity [19]. Levels of HCT increased from an average of ∼42% to ∼60% packed cell volume (PCV; **Figure 5F**). A significant increase in hemoglobin was also observed, increasing from 15 g/dl at baseline prior to the 5^th^ rhGAA injection to ∼20 g/dl ∼5 minutes after rhGAA injection (**Figure 5 G**). Oxygen saturation (pO2) dropped significantly 5 minutes after injection, accompanied by a significant increase in dissolved carbon dioxide (pCO2) indicating respiratory acidosis (data not shown) although these changes were insufficient to significantly change the blood pH within 5 minutes of rhGAA administration in the groups tested.

### GAA-/- 129SVE mice show increased coagulation on repeated rhGAA exposure

**Figure 6:**
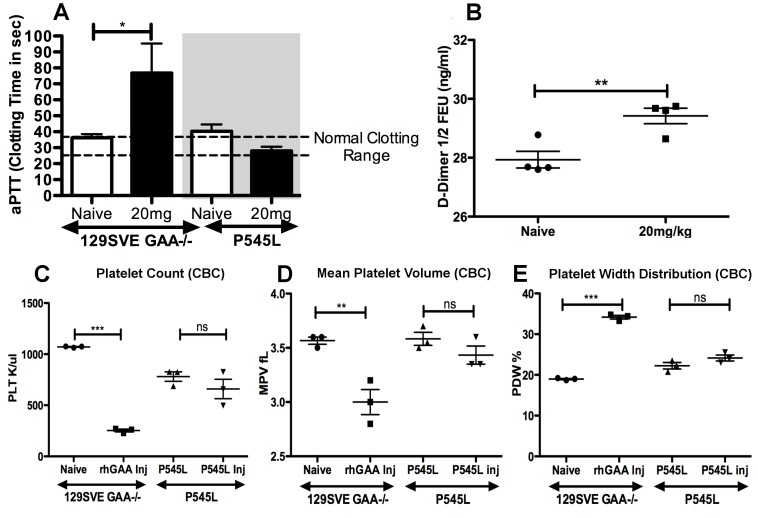
**A**) Reduction of clotting times measured in activated partial thromboplastin time (aPTT) in GAA-/- 129SVE and P545L mice. **B**) D-Dimer levels in GAA-/-129SVE naïve and rhGAA injected mice. Changes in hemorheologic values from complete blood counts in 129SVE GAA-/- mice and P545L naive and 20 mg/kg rhGAA injected mice (5 min post rhGAA injection). **C**) Platelet count, **D**) Mean platelet volume, **E**) Platelet width distribution, p<0.05 *, p<0.005 **, p<0.0005 ***, ns = not significant.

Bleeding times were measured to determine the time required for a platelet plug to form, prior to the rhGAA injection as well as 5 minutes after rhGAA injection. A very significant drop in bleeding times was observed in all six mice tested (**Figure 5 E**). Three mice survived the rhGAA administration, two of which recovered from extensive clotting when tested 5 hours after rhGAA administration (**Figure 5 E**). Additionally, an activated partial thromboplastin time (aPTT) assay indicated the occurrence of increased clotting post-rhGAA injection in GAA-/- 129SVE mice (n = 6), which was not observed in the P545L mice (n = 5; **Figure 6 A**). D-Dimer assay was done prior to and post-rhGAA 20 mg/kg injection in GAA-/- 129SVE mice (n = 4) and a significant increase in D-Dimer was observed (**Figure 6 B**).

**Figure 7:**
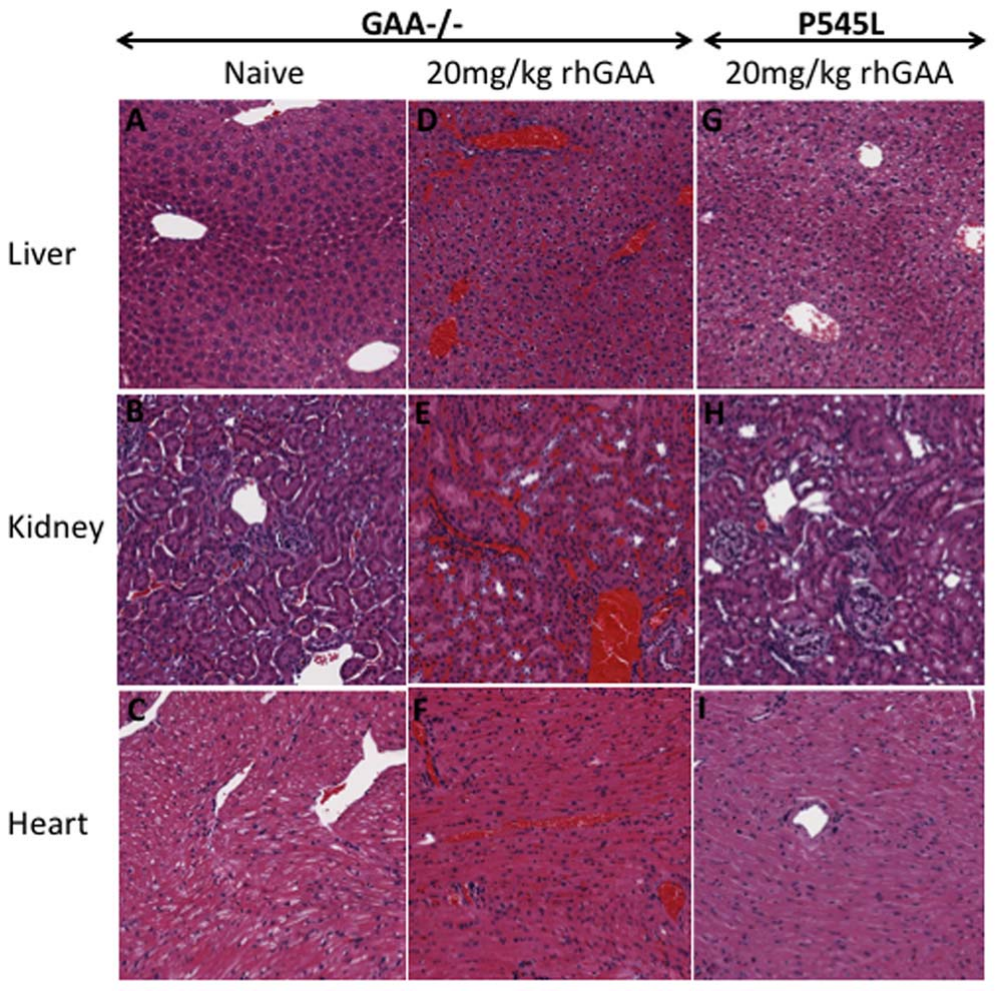
Representative examples of hematoxylin and eosin (H&E) staining of paraffin embedded sections of liver, kidney and heart (n = 3) in A-C) naïve GAA-/- 129SVE mice and D-F) 20 mg/kg rhGAA IV injected GAA-/- 129SVE indicating residual RBC, G-I) 20 mg/kg rhGAA IV injected P545L mice.

**Figure 8:**
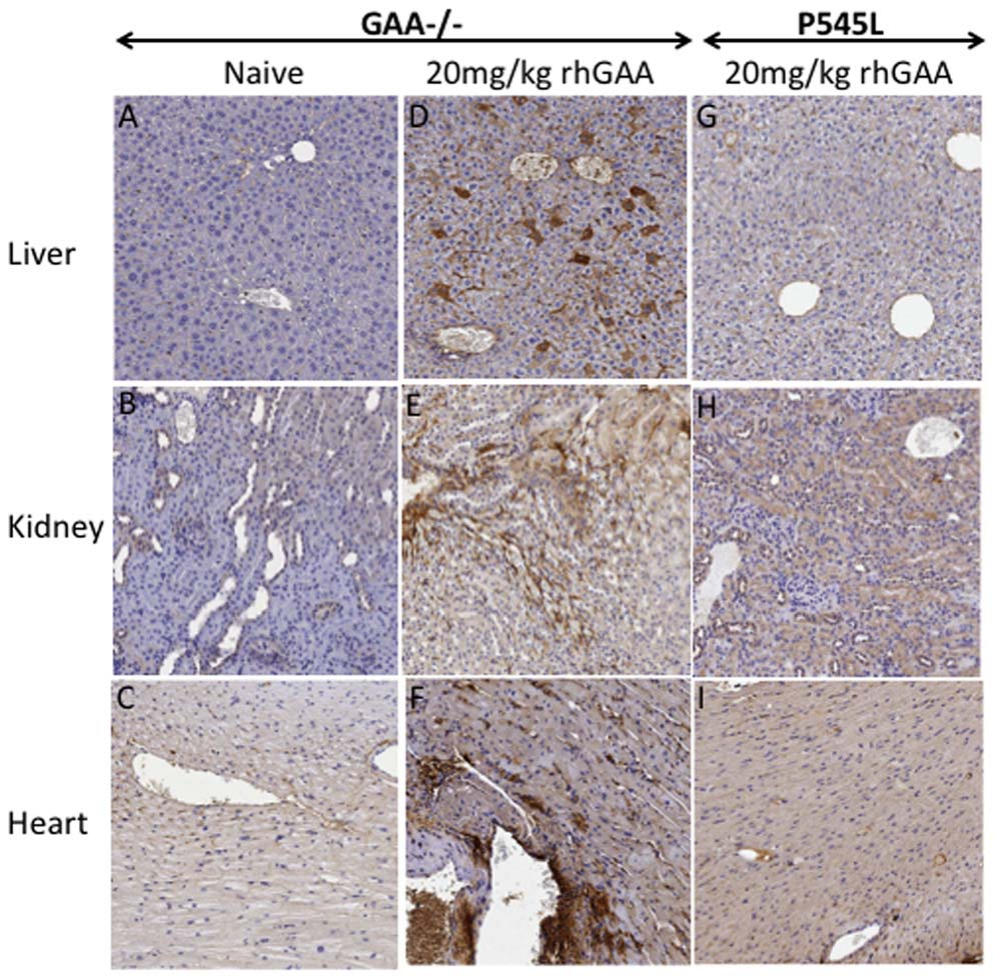
Representative examples of fibrinogen staining in paraffin embedded sections of liver, kidney and heart (n = 3) in A-C) naïve GAA-/-129SVE mice and D-F) 20 mg/kg rhGAA IV injected GAA-/- 129SVE and G-I) 20 mg/kg rhGAA IV injected P545L mice.

CBC was done on naïve GAA-/-129SVE mice and in an additional group injected with multiple rhGAA tail-vein injections. Tail bleeds were done prior to rhGAA injection as well as 5 minutes after injection. Normal platelet counts were observed in naïve mice while the group that underwent five rhGAA injections showed chronic thrombocytopenia (**Figure 6 C**). Low platelet levels were observed prior to injection as well as after injection. Increased platelet clumps were seen on blood smears of the rhGAA challenged mice (data not shown). The lowered platelet counts support results (**Figure 5 E** **, 6 A, B**) indicating the occurrence of reduced bleeding time and active clotting or hyper-coagulation within 5 minutes of exposure to rhGAA protein in GAA-/-129SVE mice. Platelet distribution width (PDW) is often indicative of active platelet release. PDW in rhGAA treated mice was significantly higher in the rhGAA-dosed group than in naïve mice (**Figure 6 E**). An increase in mean platelet volume was also observed (**Figure 6 D**). Platelet count, mean platelet volume and PDW remained unchanged after multiple 20 mg/kg rhGAA tail-vein injections in the P545L mice. Surviving mice from the 20 mg/kg GAA-/- 129SVE and P545L cohorts (n = 3) and naïve GAA-/-129 SVE mice (n = 3) were euthanized after the 6^th^ 20 mg/kg rhGAA injection. Liver, kidney and heart were sectioned and stained with hematoxylin and eosin (H&E), additional sections were stained for fibrinogen. Severe hyper-coagulation was observed in all the 20 mg/kg rhGAA treated mice for which representative samples have been shown (**Figure 7 D, E, F**). Naïve mice appear to have relatively clear interstitial spaces and blood vessels in the H&E sections (**Figure 7 A, B, C**) while the GAA injected P545L mice have minimal appearance of hyper-coagulation (**Figure 7 G, H, I**). Deposition of fibrinogen can be clearly observed in the liver, kidney and heart tissue of 20 mg/kg rhGAA treated GAA-/- 129 SVE cohort but not in the naïve GAA-/- 129 SVE and hyper-coagulation appears minimal in the 20 mg/kg treated P545L mice (**Figure 8**).

## Discussion

### Immune responses in animal models of Pompe disease

Early onset Pompe patients (CRIM-) consistently develop severe anti-rhGAA immune responses, although some CRIM+ infants and adults have also been reported with adverse reactions to rhGAA ERT [6], [20]. Low patient numbers contribute to insufficient data on the immune responses to rhGAA ERT, hence animal models of the disease are important. Studies are ongoing to develop adequate immune suppressive or immune tolerance protocols in preclinical models of Pompe disease as well as in patients [10], [21], [22], [23]. Others have attempted to develop immune tolerance protocols for Pompe disease in wt mice [24]. We have previously identified the major CD4+ T cell epitopes from rhGAA responsible for T cell responses in GAA-/-129SVE Pompe mice [11]. The use of recombinant human proteins in GAA-/- mice may cause additional immunological issues not seen in humans. Although humans develop high titer antibody responses to rhGAA ERT, coagulopathy is not known to be a common complication. The use of steroids, antihistamines and other medications during ERT makes it difficult to study ERT related coagulopathy in humans. Our current data suggest that the background of mice and the type of mutation play an important role in the development of anti-rhGAA immune responses in ERT. Studies in other protein deficiency diseases like hemophilia have also indicated the importance of the background of the mouse and the type of mutation for immunological studies [25]. Hemophilia B mice on a C3H/H3J background also show life-threatening anaphylactic reactions, associated with antibody formation after repeated intravenous enzyme administration. Similar to the Pompe mouse, immune responses are enzyme dose and Factor IX mutation dependent [26] and is associated with IgE formation. While hemophilia B mice show similarly rapid reactions and similar clinical symptoms (such as piloerection and respiratory distress [27]) it is unknown whether DIC develops in this model. Additionally, our data indicate that the use of wt BALB/c (haplotype H2^d^) and wt 129SVE mice (haplotype H2^bc^) for the study and development of tolerance protocols to rhGAA should be considered carefully as they develop less severe and altered immune responses to ERT in comparison to classic GAA knockout Pompe mouse models. Immune tolerance protocols for ERT developed solely in wt mice may not extrapolate to Pompe disease models. For our experiments, the GAA-/- C57B/6 x 129SVJ (haplotypes H2^b^ and H2^bc^) mice developed by Raben *et al*. were bred onto a pure 129SVE (haplotype H2^bc^) background to facilitate immune studies.[11], [12]

### Humoral immune responses and anaphylaxis to rhGAA ERT are antigen dose-dependent

RhGAA infusions in early onset Pompe disease produce very high titer anti-rhGAA immune responses. High titer anti-rhGAA antibodies have been found to bind up to 40% of rhGAA and hence reduce therapeutic efficacy of rhGAA in patients [6]. Our preclinical data suggest that reducing the dose of rhGAA required for successful ERT has a profound effect, resulting in reduced immune responses and reduced immune toxicities. Commercially available CHO cell-derived rhGAA is dependent on mannose-6-phosphate receptor internalization and is plagued by inefficient receptor-mediated uptake into cells [28]. Hence patients are usually dosed with 20 mg/kg or even 40 mg/kg in some cases, to achieve maximum uptake into cells for therapeutic effect. In other protein deficiency disorders like Gaucher however, the therapeutic dose is 15–120 U/kg of imiglucerase, for Fabry's disease agalsidase alpha dose is 200 µg/kg, for hemophilia A with inhibitors it is up to 400 µg/kg of Factor VIIa. [29], [30] In Hemophilia B, a dose of 15–20 U/kg of Factor IX was sufficient to cause disseminated intravascular coagulation (DIC). Others indicate the presence of IgE (Th2 dependent) in Pompe gene therapy and we have previously found the production of IL-4 while assessing Th2 immunity in response to dominant rhGAA epitopes in the GAA-/-129SVE Pompe model.[10], [11] We found that lowering the dose of rhGAA resulted in significant reduction of anti-rhGAA IgG1, IgG2a, IgG2b and IgM antibody responses in the animal models tested. Physiological responses associated with the high titer anti-rhGAA antibody responses also improved when the rhGAA antigen load was reduced. Specifically, the oxygen saturation, changes in heart rate and pulse distention improved. Effort required to breathe, represented by breath distention and breath rate also improved at reduced rhGAA doses and were concomitant with reduced immune responses at the lower rhGAA dose. High-dose rhGAA ERT was accompanied by increased mortality, piloerection and reduced activity (due to anaphylaxis) after rhGAA administration and was eliminated in the low-dose cohort. An increase in coagulation post-rhGAA injection, represented by increased aPTT, significant reduction in platelet plug formation and increased D-Dimer production, increased fibrinogen deposition, along with increased residual blood in multiple organs, possibly indicating disseminated intravascular coagulation, was determined to be associated with high-dose ERT with rhGAA in GAA-/-129SVE mice.

### Type of mutation and murine background affects the immune outcome

The type of mutation in *GAA* results in a spectrum of severity and age of onset of Pompe disease. Additionally, our data indicate that the type of mutation affects the severity of the immune responses as well as the associated immune toxicities and anaphylaxis. Wt mice (BALB/c and 129SVE background) did not produce a severe immune response or the precipitous drop in vital parameters like oxygen saturation, heart rate, breath distention or core body temperature. They also did not exhibit the external signs of anaphylaxis like piloerection and reduced movement. This is likely due to the presence of normal mouse GAA that has an 80% identity with human lysosomal GAA (Smith Walterman pairwise alignment, Protein Information Resource). The degree of identity with the injected rhGAA likely decreased the available novel antigenic peptides and hence resulted in a reduced immune response. The P545L missense mutation mice developed a low anti-rhGAA antibody response. They did not undergo anaphylaxis or observable discomfort with the multiple doses of ERT that caused severe immune responses and toxicities in the GAA-/- 129SVE mice. These P545L mice also did not develop the deleterious increase in coagulation post-rhGAA injection, unlike the GAA-/- 129SVE Pompe mice. The P545L mice are likely exposed to self-GAA antigens due to the presence of mutated human P545L protein [31], [32]. Since the P545L mutation does not affect I-A^b^-restricted CD4^+^ T cell epitopes that we previously identified, self-protein expression likely induced tolerance to GAA protein in this strain background (C57BL/6 x 129SVE) via central and/or peripheral mechanisms [11]. Other genetic factors in addition to the underlying GAA mutation may influence the response as suggested by our evaluation of different mouse strain backgrounds.

### Relevance and future directions

Anti-rhGAA antibody responses and adverse responses during rhGAA administration resulting in anaphylaxis impacts the therapeutic efficacy of ERT in Pompe disease. Immune modulation using drugs like rituximab and rapamycin are being tested with rhGAA ERT in early onset CRIM- patients.[33] Others have tested rituximab, bortizomib and methotrexate [34]. In addition, our data indicate that reducing the antigen availability will result in reduced immune responses. The use of chaperones like 1-deoxynojirimycin (DNJ) have been shown to improve the stability and promote uptake of rhGAA into cells and tissues and may be helpful in reducing the required dose of GAA for therapeutic benefit [31]. T regulatory cell epitopes are being explored for Pompe disease [35]. Others have modified GAA by adding a glycosylation-independent lysosomal targeting (GILT) tag with high affinity to the mannose 6-phosphate receptor to increase the uptake into cells, hence reducing the dose required for ERT [36]. The immune responses to rhGAA ERT in the presence of chaperones and with improved GAA lysosome targeting warrant further investigation and are likely to improve the outcome in patient populations prone to immune responses to ERT. Gains are being made in gene therapy for Pompe disease and may allow for development of alternate therapies with improved immune outcomes [37], [38].

## Supporting Information

Figure S1 **A**) Anti-rhGAA IgG2b in 1 mg/kg rhGAA injected GAA-/- 129SVE mice **B**) Anti-rhGAA IgG2b in 5 mg/kg rhGAA injected GAA-/- 129SVE mice **C**) Anti-rhGAA IgG2b in 20 mg/kg rhGAA injected GAA-/- 129SVE mice **D**) Anti-rhGAA IgG2b in 20 mg/kg rhGAA injected P545L mice **E**) Anti-rhGAA IgM in 1 mg/kg rhGAA injected GAA-/- 129SVE mice tested weekly **F**) Anti-rhGAA IgM in 5 mg/kg rhGAA injected GAA-/- 129SVE mice **G**) Anti-rhGAA IgM in 20 mg/kg rhGAA injected GAA-/- 129SVE mice **H**) Anti-rhGAA IgM response in 20 mg/kg rhGAA injected P545L mice. Arrows indicate fold decrease over corresponding 20 mg/kg time point. p<0.05 *, p<0.005 **, p<0.0005 ***, ns = not significant.
(TIF)
