# Supplementary figures and images for: Immune Responses and Hypercoagulation in ERT for Pompe Disease Are Mutation and rhGAA Dose Dependent
Source: PLoS One. 2014 Jun 4;9(6):e98336. doi: 10.1371/journal.pone.0098336 (PMC4045583; doi:10.1371/journal.pone.0098336)

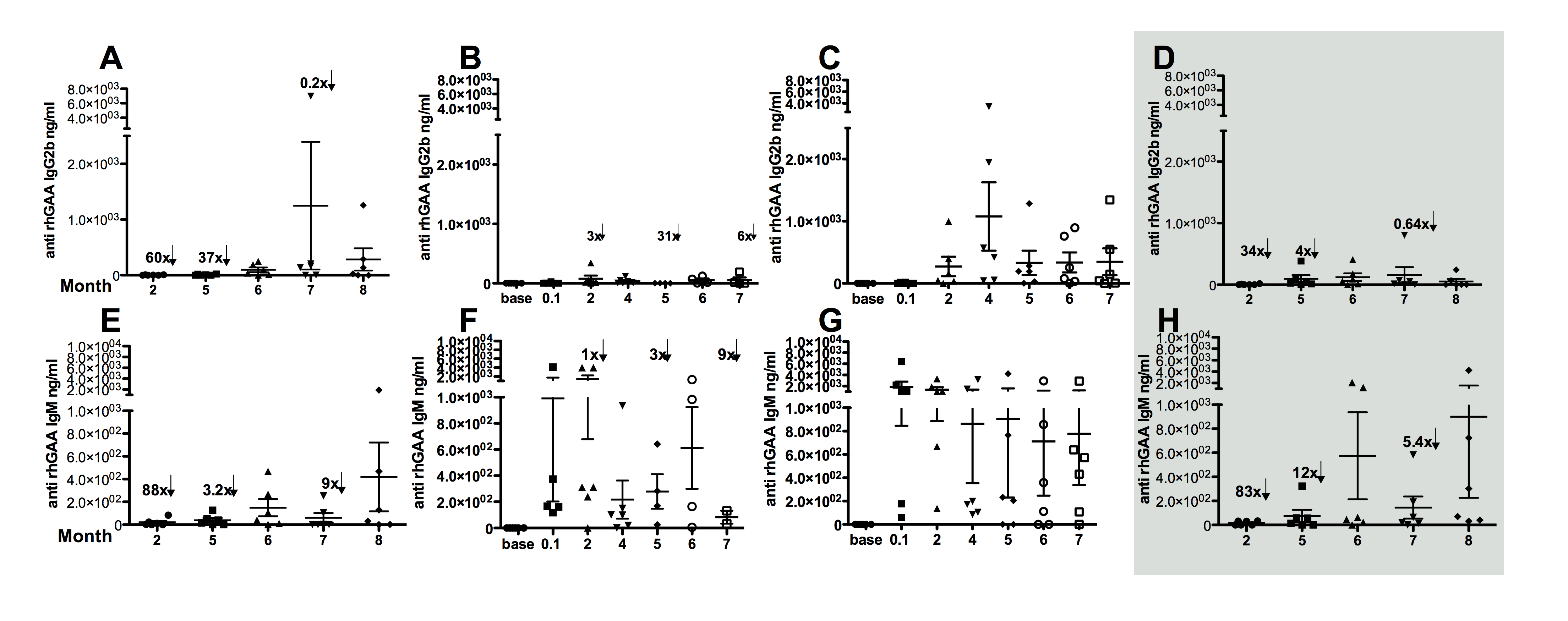

Supplement: Figure S1 — A) Anti-rhGAA IgG2b in 1 mg/kg rhGAA injected GAA-/- 129SVE mice B) Anti-rhGAA IgG2b in 5 mg/kg rhGAA injected GAA-/- 129SVE mice C) Anti-rhGAA IgG2b in 20 mg/kg rhGAA injected GAA-/- 129SVE mice D) Anti-rhGAA IgG2b in 20 mg/kg rhGAA injected P545L mice E) Anti-rhGAA IgM in 1 mg/kg rhGAA injected GAA-/- 129SVE mice tested weekly F) Anti-rhGAA IgM in 5 mg/kg rhGAA injected GAA-/- 129SVE mice G) Anti-rhGAA IgM in 20 mg/kg rhGAA injected GAA-/- 129SVE mice H) Anti-rhGAA IgM response in 20 mg/kg rhGAA injected P545L mice. Arrows indicate fold decrease over corresponding 20 mg/kg time point. p<0.05 *, p<0.005 **, p<0.0005 ***, ns = not significant. (TIF) [file pone.0098336.s001.tif]
